# Habitat effects on reproductive phenotype, pollinator behavior, fecundity, and mating outcomes of a bumble bee–pollinated herb

**DOI:** 10.1002/ajb2.1826

**Published:** 2022-03-14

**Authors:** Hao Tian, Lawrence D. Harder, Ai‐Ying Wang, Da‐Yong Zhang, Wan‐Jin Liao

**Affiliations:** ^1^ State Key Laboratory of Earth Surface Processes and Resource Ecology, Ministry of Education Key Laboratory for Biodiversity Science and Ecological Engineering Beijing Normal University Beijing 100875 China; ^2^ Department of Biological Sciences University of Calgary Calgary Alberta T2N 1N4 Canada

**Keywords:** *Aconitum*, geitonogamy, habitat differences, mate diversity, nectar, outcrossing rate, phenotypic plasticity, pollinator behavior, seed production

## Abstract

**Premise:**

Fecundity and mating outcomes commonly differ among plant populations occupying contrasting environments. If self‐pollination occurs primarily among flowers within plants, contrasting reproductive outcomes among populations must reflect environmental effects on plant‐pollinator interactions. Specifically, local conditions could affect features of plant phenotypes that influence pollinator behavior, in turn modifying plant reproductive outcomes.

**Methods:**

We compared phenotypes, pollinator abundance and behavior, and female fecundity and mating in two meadow populations and two forest populations of *Aconitum kusnezoffii* within 3 km of each other. Mating outcomes were assessed using microsatellites.

**Results:**

Meadow plants generally produced more, shorter ramets with more, larger flowers, but less nectar per flower than forest plants. These differences likely largely represent phenotypic plasticity. Individual bumble bees visited more flowers on forest plants, likely because the more abundant bees in the meadows depleted nectar availability, as indicated by briefer visits to individual flowers. Despite similar fruit set in both habitats, forest plants set more seeds per fruit. Nevertheless, meadow plants produced more seeds overall, owing to sevenfold greater flower production. Consistent with individual bees visiting fewer flowers on meadow plants, more of their seeds were outcrossed. However, the outcrossed seeds of forest plants included more male mates.

**Conclusions:**

Reproductive outcomes can vary among populations of animal‐pollinated plants as a result of differences in the availability of effective pollinators and environmental effects on plant phenotypes, and their functional consequences for pollinator behavior that governs pollen dispersal.

In angiosperms, the mating outcomes that govern population dynamics, genetically link generations, and determine population genetic structure typically arise from ecological interactions (Barrett and Harder, 2017). Environmental dependence is evident from extensive population differences in overall fruit and seed production (Knight et al., 2005; Aguilar et al., 2006) and the relative frequencies of outcrossing and selfing in self‐compatible species (Whitehead et al., 2018). In animal‐pollinated species, interpopulation variation in the fraction of outcrossed seeds is often attributed to interacting effects of the local frequency of pollinator visitation on the incidence of cross‐pollination and floral traits that enable autonomous self‐pollination when cross‐pollination is limited (Barrett et al., 1989; Kalisz et al., 2004; Moeller and Geber, 2005; Eckert et al., 2009; Leibman et al., 2018). However, differences in mating among populations may also be attributable to less‐studied causes related to pollinators' effectiveness and the quality of their visits (Hargreaves et al., 2010; Christopher et al., 2021). Most obviously, pollinators often facilitate self‐pollination within (facilitated autogamy) or among (geitonogamy) a plant's flowers (Harder and Barrett, 1995; Eckert, 2000; Owen et al., 2007), the incidence of which can vary among populations (Christopher et al., 2021). In addition, population differences in outcross‐mate diversity (Sun and Ritland, 1998; Yates et al., 2007; Zhang et al., 2017) must arise from variation in processes specifically involved in cross‐pollination.

Population differences in fecundity, facilitated selfing, and outcrossing could arise from contrasts in the local pollination environments and plant phenotypes, and their interacting effects on pollinator behavior. Relevant features of the pollination environment include the availability and density of potential mates (Delmas et al., 2015, 2016; Christopher et al., 2021), the abundance and diversity of pollen vectors (Brunet and Sweet, 2006; Delmas et al., 2015, 2016; Yin et al., 2016; Leibman et al., 2018), and the presence of other plant species that facilitate or compete for vector service (Caruso, 2000; Bell et al., 2005). In general, fecundity and cross‐mating benefit from more mates and more pollinators (Herlihy and Eckert, 2004; Christopher et al., 2021; Richardson et al., 2021), but they suffer from competition for pollinators and interspecific pollination (Caruso, 2000; Bell et al., 2005).

In animal‐pollinated species, phenotypic traits could contribute to population differences in fecundity and mating by affecting pollinator attraction and pollen exchange (Herlihy and Eckert, 2004; Dart et al., 2011; Brys et al., 2013; Leibman et al., 2018). Pollinator attraction commonly varies positively with the number of flowers that plants display simultaneously and with flower size (Conner and Rush, 1996; Ohashi and Yahara, 2001; Ishii and Harder, 2006). Once pollinators have been attracted, cross‐pollination requires that they remove pollen from visited flowers and deposit pollen from other conspecific plants on their stigmas. In general, increases in the fraction of pollen on pollinators' bodies removed by individual stigmas increase geitonogamy and decrease outcrossing and mate diversity (Harder and Barrett, 1996; Mitchell et al., 2013). This fraction, and the extent of facilitated autogamy, can vary positively with floral characteristics that control the duration of individual visits (e.g., nectar volume and concentration, ease of nectar access; Harder, 1986; Brandenburg et al., 2012) and with pollen and stigma characteristics (e.g., Harder and Johnson, 2008). Mating outcomes also vary because pollinators commonly visit more flowers on plants that display many flowers simultaneously (Ohashi and Yahara, 2001) or present abundant nectar in flowers (Hodges, 1995; Jersáková and Johnson, 2006; Brandenburg and Bshary, 2011). These responses can increase geitonogamy if pollinators visit pollen‐receiving flowers after pollen‐presenting flowers on the same plant (Harder et al., 2000; Karron et al., 2004). For clonal species, geitonogamy can occur within or among physical individuals (ramets) of the same genetic individual (genet), and thus can vary positively with ramet number (Eckert, 2000; Vallejo‐Marín et al., 2010; Hu et al., 2015). Given these influences, differences among populations in features of the pollination environment and/or plant characteristics that affect pollinator behavior and pollen exchange could cause corresponding differences in mating outcomes.

Floral and plant traits that affect pollination and mating can vary among populations genetically (Herlihy and Eckert, 2007; Ellis and Johnson, 2009; Zhao and Huang, 2013) and plastically (Elle and Hare, 2002; Morales et al., 2010; Meindl et al., 2013; Dai et al., 2017; Christopher et al., 2021). Most obviously, local adaptation to the dominant effective pollinator can generate pollination ecotypes that differ primarily in floral traits (Van der Niet et al., 2014). Local adaptation and plastic responses to abiotic conditions may also affect mating outcomes. Favorable conditions for plant growth are particularly germane if larger plants produce more inflorescences and ramets with more, larger flowers (e.g., Kilkenny and Galloway, 2008; Celis et al., 2019). Also relevant are conditions responsible for population differences in nectar production per flower (e.g., Brink and de Wet, 1980; Gijbels et al., 2014), given the latter's influences on pollinator attraction, visit duration, and number of flowers visited per pollinator (Klinkhamer and van der Lugt, 2004; Brandenburg and Bshary, 2011).

Despite evidence that population differences in fecundity and mating can arise from diverse environmental influences on pollination environments and plant phenotypes, the relative contributions of these influences have seldom been considered simultaneously. Here, we assess the consequences of population differences in plant phenotypes and pollinator abundance for pollinator behavior and plant female fecundity, outcrossing, and male‐mate diversity. We specifically compare four populations of *Aconitum kusnezoffii* Rchb. (Ranunculaceae), a clonal perennial herb, pollinated mostly by a single bumble‐bee species, *Bombus ignitus* Smith. Two populations occupied open meadows and two occurred in deciduous forests, all within 3 km of each other. The contrasting abiotic conditions (e.g., light, soil moisture, and nutrients) between open sites and forest understory greatly influence plant growth and phenotypes (Galloway and Etterson, 2009; Atlan et al., 2015; Celis et al., 2019), possibly affecting genet and ramet floral displays and floral traits (e.g., size and nectar production). Specifically, for species capable of growth and reproduction in both shaded and open environments, open‐grown individuals generally grow larger and produce more flowers (Galloway and Etterson, 2009; Atlan et al., 2015). In addition, because of differences in temperature and light intensity, bumble‐bee pollinators are generally more abundant in meadow populations than in forest populations (see Harder, 1985; Kilkenny and Galloway, 2008; Pengelly and Cartar, 2010; Cao et al., 2017; Richardson et al., 2019). Consequently, nectar standing crops may often be larger in flowers of forest plants, owing to longer intervals between flower visits. If so, individual bees in forest populations should spend more time visiting individual flowers and visit more flowers per genet. These responses could increase facilitated autogamy and geitonogamy, depending on the effectiveness of the separation of female and male floral phases in this protandrous species. The likely population effects on cross‐pollination, seed production, and male‐mate diversity are less predictable, as they depend on the details of the pollinator responses to floral and inflorescence traits and associated pollen‐dispersal characteristics (Harder et al., 2004; Leibman et al., 2018; Minnaar et al., 2019) and the effects of inbreeding on seed development (Maki, 1993; Herlihy and Eckert, 2002; Owen et al., 2007; Delmas et al., 2014; Van Etten et al., 2015). In addition to assessing these possible environment‐performance associations, we consider the extent to which the phenotypic differences among the study populations represent local adaptation and/or plasticity.

## MATERIALS AND METHODS

### Study species and sites

*Aconitum kusnezoffii* is a clonal herb that predominantly outcrosses (Liao et al., 2009; Hu et al., 2015). Clonal propagation via tubers creates clumped genets, usually comprising multiple reproductive ramets (hereafter, “plant” refers to a genet, not a ramet, because selfing and outcrossing involve genetic individuals). Flowering ramets produce a terminal inflorescence with ≤35 flowers (median = 9) and ≤25 lateral inflorescences (median = 8) with ≤20 flowers (median = 4). Flowering occurs from late July until mid‐September. Individual flowers are protandrous, with 4–5 d of pollen exposure preceding 2 d of stigma receptivity (Liao et al., 2009). Because female and male phase overlap in only ~5% of flowers (Liao et al., 2009), protandry limits within‐flower self‐pollination (Hu et al., 2015). Instead, most self‐pollination occurs among flowers (geitonogamy), primarily within, rather than among, ramets (Hu et al., 2015). Self‐pollinated flowers produce fewer seeds than cross‐pollinated flowers, indicating pre‐dispersal inbreeding depression (Hao et al., 2012).

The study populations were located in Xiaolongmen National Forest Park (39°57′32.1″N, 115°27′03.8″E), West Beijing, China. Two lower, meadow populations (population M1, 1034 m; M2, 1075 m) were separated by ~200 m along a stream in the same valley. These populations were fully sunlit from 0830 to 1630 hours, except that M2 was shaded by an adjacent mountain for ~3 h during midmorning. The other two populations occupied the understory of *Populus cathayana*–*Juglans manshurica* forests in separate valleys at slightly higher elevation and were separated by ~800 m (population F1, 1188 m; F2, 1220 m). Both forest populations were shaded during the flowering period of *A. kusnezoffii*, although the forest occupied by F1 was less dense, allowing more sunflecks. Flowering began ~1 wk earlier in the meadow populations than in the forest populations, likely owing to the habitat and elevation differences. Some of these populations have been studied previously in other contexts (M1, F1: Liao et al., 2009; M1: Hao et al., 2012; M1, F1: Hu et al., 2015; M1: Ge et al., in press).

This study was conducted during 2013 and 2015 (using different plants). Sampling during 2013 assessed reproductive traits, the number of flowers probed by individual bees on the first inflorescence visited per genet, and female reproductive outcomes, including mating‐system characteristics. During 2015, we conducted more detailed sampling of nectar production and pollinator behavior but did not measure plant traits or reproduction.

### Plant traits

We measured vegetative and floral traits for 30 genets in each population. Distinct clumps of ramets separated by ≥2 m from other clumps were considered as individual genets. Liao et al. (2009) found all ramets within each of 10 such clumps with >20 ramets to have identical genotypes for six polymorphic allozyme loci. Length traits were measured with a ruler.

#### Genet size and ramet height

As a measure of genet size (clonality), we counted all flowering ramets for each genet. To quantify ramet height, we measured the distance from the ground to the top of the uppermost open flower for each flowering ramet (±0.5 cm).

#### Flower production

For up to five flowering ramets per genet (fewer if five were not available), we counted the lateral inflorescences. For the terminal inflorescence and one middle lateral inflorescence, we also counted the flowers and measured flower size as the long axis of the perianth (±0.1 mm) of each of three scattered open flowers.

#### Nectar

During mid‐flowering, we measured nectar volume with 10 µL capillary tubes and sugar concentration of four female‐ and four male‐phase flowers on each genet with a sucrose refractometer (Atago MASTER‐53Pα, Bellevue, Washington, USA). From 0800 to 0830 hours during mornings 24 h prior to measurement, we enclosed flowers in mesh bags. Because the bumble bees that visit *A. kusnezoffii* forage only during daylight, the nectar accumulated in bagged flowers may represent up to 36 h of production. Using the volume and concentration measurements, we calculated the total sugar mass for each flower following Bolten et al. (1979) and Galetto and Bernardello (2005).

### Pollinator behavior

To determine whether pollinator abundance and visitation differed between meadow and forest plants, we observed bumble bees on twelve (2013) or eighteen (2015) genets per population during mid‐flowering. Visiting bumble bees foraged primarily for nectar. We counted all open flowers and observed all flowering ramets of each clone for 30 min between 0930 and 1530 hours on sunny days. During 2013, we recorded flower visits to the first inflorescence visited per genet by individual bees with digital video cameras (total observation time = 24.0 genet h). While viewing these recordings, we measured the duration of each flower visit with a stopwatch. During 2015, observers used voice recorders to record the number of pollinators visiting focal genets, the number of ramets and total number of flowers visited per genet, and the duration of visits to individual flowers (total observation time = 40.5 genet h).

Counts of the numbers of displayed flowers (*f*) and the flowers visited by bee *i* (*v*_*i*_) for individual genets allowed estimation of the probability that each flower received at least one visit by the *B* bees that visited a genet during its 30 min observation period:

$$p=1-\prod_{i}^{B}\left(1-\frac{v_{i}}{f}\right).$$

The term in parentheses is the proportion of flowers that bee *i* did not visit, if flowers were visited independently, and the product estimates the probability that none of the *B* bees visited a specific flower.

### Female reproductive outcomes

For plants on which we measured floral traits that retained undamaged inflorescences, we recorded whether each flower produced a fruit and collected intact fruits (~90% of the total) several weeks after flowering, as they matured. For up to six fruits per ramet, we recorded the number of carpels with seeds, and for those carpels we counted all ovules and seeds. The six fruits included three from the terminal inflorescence and one each from three lateral inflorescences on the same ramet. Fewer than six fruits were collected per ramet if six were not available (owing to limited fruit number or insect damage).

We randomly selected 22 seeds per genet for 20 of the genets per population used for fecundity assessment to estimate mating‐system parameters with microsatellite markers (Ge et al., 2016). We also collected pieces of two leaves from each genet, which were dried in silica gel, to characterize maternal genotypes. In preparation for DNA extraction, seeds were soaked for 12 h and their seed coats were removed. Total genomic DNA was extracted using a plant genomic DNA extraction kit (Tiangen, Beijing, China). Five SSR loci (GenBank accession nos. KU302084, KU302087, KU302088, KU302091, KU302095) were assayed with a 3730 Genetic Analyzer (Applied Biosystems, Thermo Fisher Scientific, Grand Island, New York, USA; for details, see Ge et al., 2016). Genotypes were scored with GeneMapper version 3.2 (Applied Biosystems).

We quantified mating outcomes based on maternal and seed genotypes using Colony version 2.0.6.4 (Jones and Wang, 2010; Wang et al., 2012). Colony infers the probability that each seed was outcrossed and identifies its (possibly unknown) father. Whether a seed was outcrossed or selfed was identified with high certainty: the probability of outcrossing was either <0.01 (i.e., selfed) or >0.99 (i.e., outcrossed) for 96.5% of the 1748 genotyped seeds. The outcrossing probabilities for individual seeds were used in subsequent statistical analysis to estimate the proportion of seeds in maternal families that were outcrossed (female outcrossing rate). The paternal identities inferred by Colony were used to quantify male‐mate number for each maternal genet, given the number of assayed outcrossed seeds. We also used Maki's (1993) method to estimate the mean proportions of outcrossed zygotes for meadow and forest plants, based on the proportion of outcrossed seeds and Hao et al.'s (2012) estimate of pre‐dispersal inbreeding depression for population M1 (*δ* = 0.315; same estimate used for all populations).

### Statistical methods

Statistical analyses of pollinator behavior, plant phenotypes, and reproductive outcomes involved generalized linear or generalized linear mixed models (Stroup, 2013), as implemented in the Glimmix procedure of SAS/STAT version 15.1 (SAS Institute, 2018) or the glmmTMB function of the package glmmTMB version 1.1.2.3 in R version 4.1.1 (analyses of the numbers of flowering ramets and visited ramets). All analyses employed maximum likelihood methods to estimate parameters. For mixed models, this involved Laplace approximation of the integral of random effects (Stroup, 2013). All analyses considered sampling distributions and link functions appropriate for the characteristics of the dependent variables (identified in the footnotes of Tables 1 and 2). Analyses of total bee visits per genet and the numbers of ramets and flowers visited per bee did not include ramet or total flower number as covariates, because we focused on the plant perspective (specifically the opportunity for geitonogamy), not the bee perspective.

**Table 1:** Results of linear and generalized linear mixed models assessing sources of variation in features of genets, ramets, nectar, pollinator behavior, and mating outcomes in *Aconitum kusnezoffii*

Dependent variable	Effect		
	Habitat	Population(habitat)	Genet(hab pop)
Genet characteristics			
Flowering ramets^a^ ^ (tN^ B ^)^	$\chi_{1}^{2}$ = 123.36***	$\chi_{2}^{2}$ = 31.48***	
Ramet characteristics			
Ramet height^a^ ^ (N)^	$\chi_{1}^{2}$ = 86.25***	$\chi_{2}^{2}$ = 25.51***	LR_1_ = 115.4***
Lateral inflorescences^a^ ^ (P)^	$\chi_{1}^{2}$ = 47.38***	$\chi_{2}^{2}$ = 14.63**	LR_1_ = 133.0***
Nectar characteristics			
Nectar volume^a^ ^ (LN), ^ b	$\chi_{1}^{2}$ = 166.4***	$\chi_{2}^{2}$ = 13.65**	LR_1_ = 10.10***
Nectar concentration^a^ ^ (*β*)^	$\chi_{1}^{2}$ = 1.16	$\chi_{2}^{2}$ = 38.40***	
Nectar sugar mass^a^ ^ (LN)^	$\chi_{1}^{2}$ = 68.94***	$\chi_{2}^{2}$ = 2.69	
Pollinator behavior			
Bees per genet^a^ ^ (QP), ^ c	$\chi_{1}^{2}$ = 19.41***	$\chi_{2}^{2}$ = 5.53	
Ramets visited^a^ ^ (tN^ B ^), ^ c	$\chi_{1}^{2}$ = 4.16*	$\chi_{2}^{2}$ = 7.10*	LR_1_ = 4.18*
Total flowers visited^a^ ^ (N^ B ^), ^ c	$\chi_{1}^{2}$ = 4.27*	$\chi_{2}^{2}$ = 5.56	LR_1_ = 0.84
Visit duration per flower^a^ ^ (LN), ^ d	$\chi_{1}^{2}$ = 23.61***	$\chi_{1}^{2}$ = 0.32	LR_8_ = 271.9***
Mating system			
Female outcrossing rate^a^ ^ (B)^	$\chi_{1}^{2}$ = 8.44**	$\chi_{2}^{2}$ = 6.95*	LR_1_ = 100.6***
Male mates^a^ ^ (QP), ^ e	$\chi_{1}^{2}$ = 5.42*	$\chi_{2}^{2}$ = 7.51*	

^a^ *β* = beta distribution, logit link function; B = binomial distribution, logit link function; LN = lognormal distribution, identity link function; N = normal distribution, identity link function; NB = negative‐binomial distribution, ln link function; tNB = zero‐truncated negative‐binomial distribution, ln link function; P = Poisson distribution, ln link function; QP = quasi‐Poisson distribution, ln link function.

^b^ This analysis also included year ($\chi_{1}^{2}$ = 34.59***), year × habitat ($\chi_{1}^{2}$ = 2.24), and year × population(habitat) ($\chi_{2}^{2}$ = 2.02).

^c^ This analysis also included genet(sampling period); bees per genet, LR_1_ = 0.64; number of ramets visited, LR_1_ = 4.92*; and total flowers visited, LR_1_ = 0.84.

^d^ This analysis also included year ($\chi_{1}^{2}$ = 22.49***), year × habitat ($\chi_{1}^{2}$ = 1.54), year × population(habitat) ($\chi_{1}^{2}$ = 0.03), and bee(hab pop genet) (LR_1_ = 277.3***), as well as separate estimates of among‐genet variation for each year × population combination (test of among‐population homogeneity, LR_7_ = 30.80***). The test of among‐genet variation presented in the table includes both the overall average and the year‐population heterogeneity, as represented in the eight degrees of freedom.

^e^ This analysis also included the ln(number of outcrossed seeds) ($\chi_{1}^{2}$ = 56.67***).

* *P* < 0.01

** *P* < 0.01

*** *P* < 0.001.

**Table 2:** Results of generalized linear mixed models characterizing variation in the number and size of *Aconitum kusnezoffii* flowers, fruit set, and seeds per fruit

Effect	Flower number^a^	Flower size^b^	Fruits per flower^c^	Seeds per fruit^a^
Habitat	$\chi_{1}^{2}$ = 4.39*	$\chi_{1}^{2}$ = 13.00***	$\chi_{1}^{2}$ = 0.01	$\chi_{1}^{2}$ = 10.23**
Population(habitat)	$\chi_{2}^{2}$ = 5.45	$\chi_{2}^{2}$ = 2.10	$\chi_{2}^{2}$ = 5.57	$\chi_{2}^{2}$ = 3.18
Inflorescence type	$\chi_{1}^{2}$ = 854.7***	$\chi_{1}^{2}$ = 829.6***	$\chi_{1}^{2}$ = 69.41***	$\chi_{1}^{2}$ = 41.94***
Inf type × habitat	$\chi_{1}^{2}$ = 31.78***	$\chi_{1}^{2}$ = 118.4***	$\chi_{1}^{2}$ = 2.11	$\chi_{1}^{2}$ = 0.01
Inf type × pop(hab)	$\chi_{2}^{2}$ = 16.83***	$\chi_{2}^{2}$ = 14.91***	$\chi_{2}^{2}$ = 8.96*	$\chi_{2}^{2}$ = 11.98**
ln(total ovules)				$\chi_{1}^{2}$ = 712.4***
ln(assayed carpels)				$\chi_{1}^{2}$ = 22.80***
Genet(hab pop)	LR_1_ = 56.59***	LR_1_ = 167.2***	LR_1_ = 7.28**	LR_1_ = 84.66***
Ramet(hab pop gen)	LR_1_ = 43.30***	LR_1_ = 168.9***	LR_1_ = 75.74***	LR_1_ = 72.30***

^a^ Poisson distribution, ln link function.

^b^ Normal distribution, identity link function.

^c^ Overdispersed binomial distribution, logit link function.

* *P* < 0.05

** *P* < 0.01

*** *P* < 0.001.

Independent variables considered in the analyses represented general and specific environmental influences. All analyses included habitat (meadow or forest) and population nested within habitat (denoted as population(habitat)) as fixed factors. Nectar volume and visit duration per flower were measured during both 2013 and 2015. Because these years were not drawn randomly from a larger set of possible years and only two years were sampled, year was treated as a fixed effect, which was crossed with habitat and population (habitat). Some analyses also included covariates to account for variation in sampling intensity (seeds/fruit analysis, covariates ln(carpel number) and ln(total ovule number); outcrossed male‐mate number analysis, covariate ln(number of outcrossed seeds)). Random effects included genet nested within population for analyses of characteristics measured for multiple ramets or flowers per genet, and ramet nested within genet for analyses of measurements of multiple flowers per ramet. Analysis of aspects of bee behavior (except visit duration) included genet(sampling period) as a random effect, and that of visit duration also included bee(genet).

Because all analyses considered the effect of each independent variable after accounting for the effects of other independent variables, we present results to illustrate these partial effects. For categorical factors, we present marginal (least‐squares) means (Milliken and Johnson, 1984). To illustrate the relation of male‐mate number to the number of genotyped outcrossed seeds per genet, we present observations adjusted for differences among habitats and populations. These values were calculated by adding an observation's residual to its mean predicted by the overall regression equation. Presentation of all results for non‐normal dependent variables involved back‐transformation of estimates from the link function, which resulted in asymmetric standard errors.

## RESULTS

### Plant and floral characteristics

**Figure 1:**
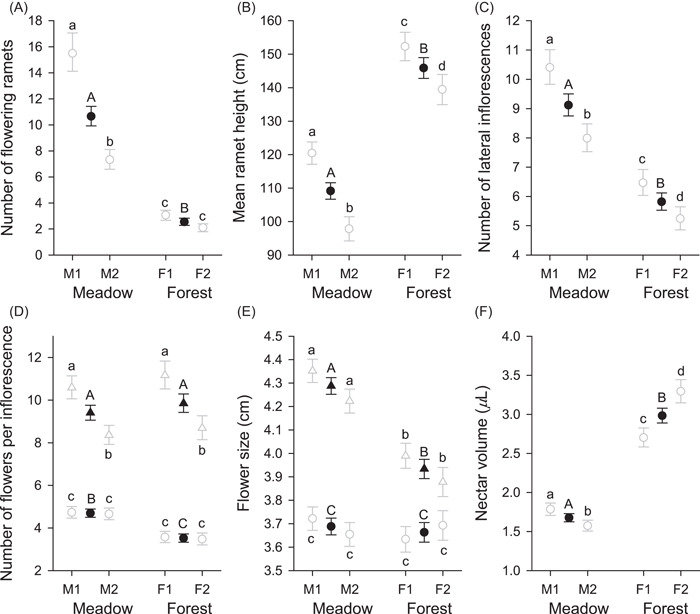
Comparisons of genet, ramet, and flower characteristics of *Aconitum kusnezoffii* between meadow and forest populations, including (A) number of flowering ramets, (B) mean ramet height, (C) number of lateral inflorescences per ramet, (D) number of flowers, and (E) mean flower size (length of the longest axis) for terminal (triangles) and lateral (circles) inflorescences, and (F) nectar volume per flower after bagging for 24 h. Values are means ± SE, based on observations from 2013, except for nectar volume, which was also sampled during 2015. Filled symbols indicate habitat means and open symbols indicate population means. Habitat means associated with different capital letters differ statistically, as do population means for the same habitat associated with different lowercase letters (*α* = 0.05). See Tables 1 and 2 for overall statistical results

Most measured phenotypic characteristics of *A. kusnezoffii* genets and ramets differed extensively between meadow and forest and, to a lesser extent, between populations within habitats (Figure 1A–D; Tables 1 and 2). On average, genets of meadow plants produced 4.2× more flowering ramets than forest plants (Figure 1A). Although flowering ramets of meadow genets were 25.2% shorter than those in forests (Figure 1B), they produced 56.7% more lateral inflorescences (Figure 1C). In addition, although flower number of terminal inflorescences did not differ between habitats, lateral inflorescences of meadow plants produced 33.2% more flowers than those of forest plants (Figure 1D). Overall, the greater production of ramets, lateral inflorescences, and flowers per lateral inflorescence resulted in meadow genets producing an average of 555.7 flowers, compared to 77.0 flowers for forest genets. In general, when characteristics differed between populations occupying the same habitat, plants in population M1 were larger than those in M2, and plants in population F1 were larger than those in F2 (Figure 1A–D). In addition to these differences, ramet characteristics varied among genets within populations and flower production varied among ramets within genets (Tables 1 and 2).

Flower size also differed between habitat types and populations, and between terminal and lateral inflorescences within ramets (Table 2). As with flower number, meadow plants produced larger flowers than forest plants, although this effect was apparent for terminal inflorescences but not lateral inflorescences (Figure 1E). Specifically, flowers on terminal inflorescences of meadow genets were 9.0% larger than those of forest plants. Flower size also varied extensively among genets within populations and among ramets within genets (Table 2), but not between populations within habitat types (Figure 1E).

Despite forest plants producing fewer, smaller flowers, their flowers contained more nectar sugar than those of meadow plants after being bagged for 24 h (Table 1). This difference likely reflects the 78.0% greater nectar volume per flower of forest plants (Figure 1F; Table 1), given that nectar concentration measured during 2015 did not differ statistically between habitat types (Table 1; overall mean = 0.442 mg/μL, lower SE = 0.0050, upper SE = 0.0051). Nectar volume also differed between years, being 30.1% greater during 2015 than during 2013. By contrast, nectar volume did not differ between female‐ and male‐phase flowers (*P* > 0.15 for main effect and all interactions, results not shown). During 2015, nectar volume and concentration differed between populations within habitat types, with more, less concentrated nectar in M1 than M2 and less, more concentrated nectar in F1 than in F2 (Figure 1F). Because of the contrasting volume and concentration patterns, total sugar mass did not vary among populations within habitat types (Table 1). Nectar volume also varied statistically among genets within populations (Table 1; 2013 only; no replicate measurements of genets during 2015).

### Pollinator abundance and behavior

**Figure 2:**
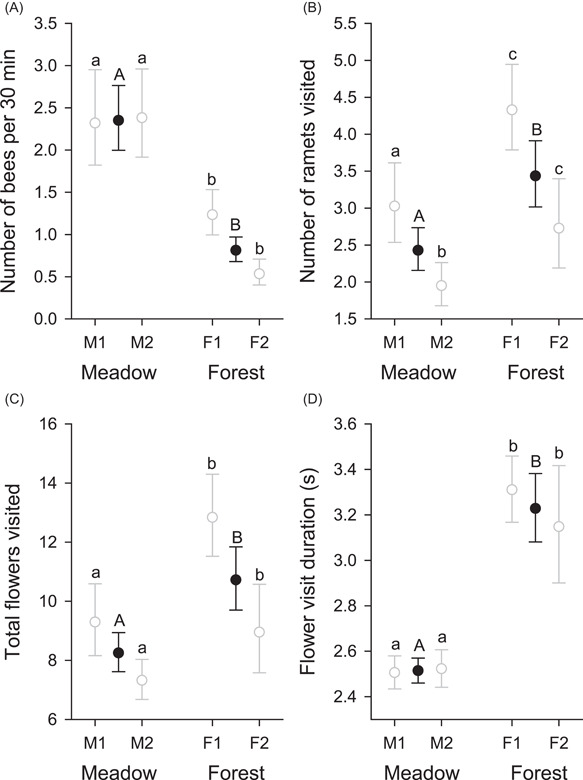
Characteristics of visitation of *Aconitum kusnezoffii* genets by *Bombus ignitus*, including (A) number of bees visiting individual genets during 30 min observation periods; (B) and (C) numbers of ramets and flowers, respectively, visited per bee per genet; and (D) duration of individual flower visits. Values are means ± SE, based on observations from 2015, except for visit duration, which was also sampled during 2013. Filled symbols indicate habitat means and open symbols indicate population means. Habitat means associated with different capital letters differ statistically, as do population means for the same habitat associated with different lowercase letters (*α* = 0.05). See Table 1 for overall statistical results

The behavior of *B. ignitus* while visiting *A. kusnezoffii* differed between meadow and forest plants (Figure 2), but not generally between populations within habitat types (Table 1). During 30 min observations in 2015, almost 3× more bees visited meadow genets than forest genets (Figure 2A). By contrast, individual bees on forest genets visited 33.6% more ramets (Figure 2B) and 29.9% more flowers (Figure 2C) than those visiting meadow genets. Habitat differences in the total number of flowers visited per genet by individual bees primarily reflect differences in ramet visitation, as the number of flowers visited per genet did not differ between habitats ($\chi_{1}^{2}$ = 0.05, *P* > 0.8) in an analysis that also included ln(number of ramets visited) ($\chi_{1}^{2}$ = 180.59, *P* < 0.001; partial regression coefficient, *b* ± SE = 0.796 ± 0.059). Because individual bees visited more flowers on forest genets but meadow genets produced many more flowers, the probability of individual flowers receiving at least one visit during a 30 min observation period did not differ between habitats ($\chi_{1}^{2}$ = 0.01, *P* > 0.9) or between populations within habitats ($\chi_{2}^{2}$ = 1.04, *P* > 0.3; generalized linear model, beta distribution, logit link function; overall mean = 0.339, lower SE = 0.0167, upper SE = 0.0171). Observation period within population affected the number of ramets visited, but not the numbers of bees per genet or total number of flowers visited (Table 1).

The duration of individual flower visits differed consistently between years and habitats (no interaction), but not between populations within habitats (Table 1). On average, bees spent 27.6% longer probing flowers during 2015 than during 2013. Overall, bees visited flowers 28.4% longer on forest plants than on meadow plants (Figure 2D). These results mirror the differences in nectar volume between years and habitats (e.g., compare Figures 1E and 2D). In addition to these effects, visit duration varied among individual bees and among genets within populations, especially for forest plants during 2015 (Table 1).

### Female reproductive outcomes

**Figure 3:**
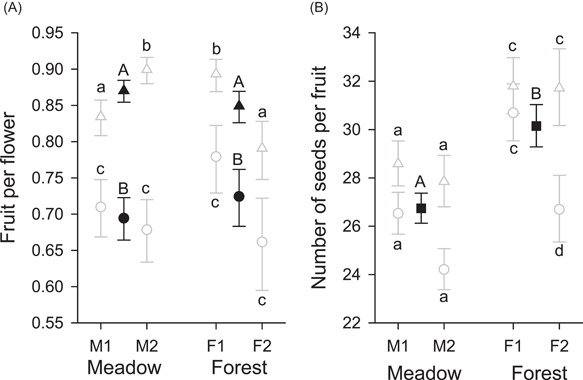
Mean (±SE) fruits per flower (A) and seeds per fruit (B) for terminal (triangles) and lateral (circles) inflorescences of *Aconitum kusnezoffii* during 2013. Filled symbols indicate habitat means and open symbols indicate population means. Habitat means associated with different capital letters differ statistically, as do population means for the same habitat associated with different lowercase letters (*α* = 0.05). The analysis of seeds per fruit also accounted for among‐flower differences in the number of assayed carpels and the number of ovules per flower. See Table 2 for overall statistical results

Fruit set per flower and seeds per fruit of the *A. kusnezoffii* genets sampled during 2013 exhibited somewhat different patterns of variation (Figure 3). A higher proportion of flowers set fruit on terminal inflorescences (86.0%) than on lateral inflorescences (71.0%; Table 2 and Figure 3A) and flowers on terminal inflorescences set fewer fruits in population F2 than in F1 (inflorescence type × population(habitat) interaction). Overall, fruit set did not differ between habitats or between populations within habitats (Table 2; Figure 3A). By contrast, seed number per fruit did differ between habitats, being 12.7% higher for forest plants than for meadow plants, after accounting for a positive effect of ln(ovule number) (*b* ± SE = 1.254 ± 0.047) and a negative effect of the number of assayed carpels (*b* ± SE = − 0.234 ± 0.049) (Figure 3B). Seeds per fruit did not vary overall among populations within habitats, although flowers on lateral inflorescences produced fewer seeds in population F2 than in F1 (Table 2, inflorescence type × population(habitat) interaction). Both fruit set and seeds per fruit varied among genets within populations and among ramets within genets (Table 2). Based on the aggregate contributions of flower production, fruits per flower, and seeds per fruit, total female fecundity (seed production) differed sixfold between habitats (meadow > forest), and ~2.5‐fold between populations within habitats (M1 > M2, F1 > F2). Given the comparatively modest variation in fruit set and seeds per fruit described above, much larger differences in total fecundity primarily reflect the substantial differences in total flower production per genet (Figure 1A,C,D).

**Figure 4:**
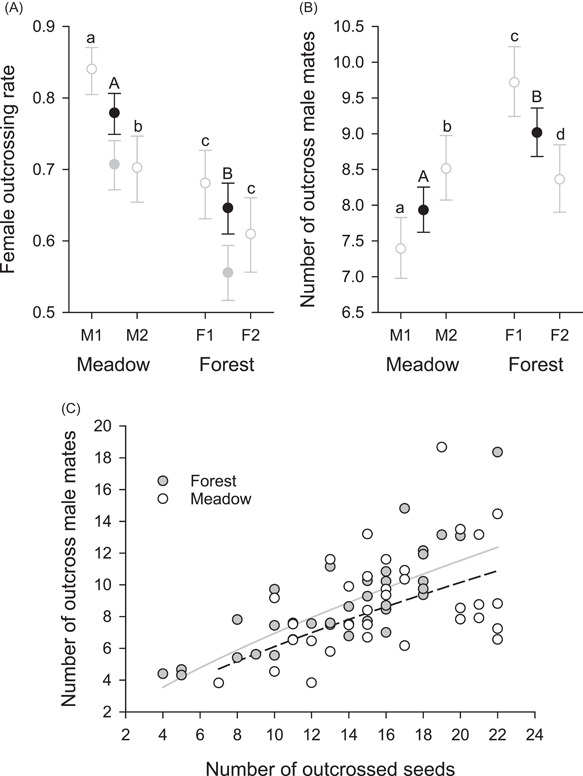
Mating outcomes for genets of *Aconitum kusnezoffii* during 2013, including (A) mean (±SE) proportions of outcrossed zygotes (gray symbols) and seeds (black and white symbols), (B) mean (±SE) number of male mates, and (C) the relation of mate number to the number of genotyped outcrossed seeds for individual plants. In A and B, habitat means associated with different capital letters differ statistically, as do population means for the same habitat associated with different lowercase letters (*α* = 0.05). In C, the dashed and solid curves represent the meadow and forest genets, respectively, with different intercepts but the same ln‐ln slope. See Table 1 for overall statistical results

Although forest plants produced more seeds per fruit, a smaller fraction of those seeds (0.646) were outcrossed than for meadow plants (0.779; Figure 4A, black symbols; Table 1). The female outcrossing rates estimated for seeds exceeded the estimated proportions of cross‐fertilized zygotes by 10%–16% (Figure 4A, gray symbols) owing to pre‐dispersal inbreeding depression. The fraction of outcrossed seeds also differed between the two meadow populations (M2 > M1), but not between the forest populations. In addition, female outcrossing rate varied extensively among genets within populations (Figure 4A, white symbols; Table 1).

Overall, the outcrossed seeds assayed per genet (mean = 14.3) were sired by an average of 8.7 male mates (lower SE = 0.31, upper SE = 0.32). The number of male mates inferred for individual genets varied positively with the number of genotyped outcrossed seeds (Figure 4C). After accounting for this sampling variation, mean male‐mate diversity differed between habitats (Table 1), being ~14% greater for forest genets than for meadow genets (Figure 4B,C). Mean male‐mate diversity also differed between populations within habitats (Table 1, Figure 4B).

## DISCUSSION

Seed production and mating patterns of *A. kusnezoffii* differed considerably between meadow and forest populations in association with contrasting plant characteristics and pollinator abundance and behavior. Compared to forest genets, meadow genets generally produced more ramets with more, larger flowers (Figure 1A,C–E). Based on the flower‐number difference alone, bumble bees would be expected to visit more flowers per genet on meadow plants (Ohashi and Yahara, 2001), resulting in a lower female outcrossing rate (Harder and Barrett, 1996). Instead, bees visited fewer ramets and fewer flowers on meadow genets than on forest genets (Figure 2B,C). Correspondingly, a larger fraction of seeds produced by meadow genets were outcrossed than for forest genets (Figure 4A). However, seeds in fruits of meadow genets were sired by fewer different pollen donors than those of forest genets, after accounting for genotyping effort (Figure 4B,C). Bees also spent less time visiting individual flowers of meadow genets than those of forest genets (Figure 2D), which may have contributed to the fewer seeds in fruits of meadow plants (Figure 3B). Nevertheless, meadow genets produced more seeds overall because although fruit set did not differ between habitats, meadow genets produced 7× more flowers than forest plants. We now consider the processes that likely link these results and the general relevance of environmental effects on plant phenotypes and pollinator behavior for population differences in reproductive outcomes of animal‐pollinated plants.

### Phenotypic variation

Plants influence their fecundity and mating outcomes via ramet, inflorescence, and floral traits that mediate their interactions with pollen vectors and autonomous self‐pollination (Barrett, 2002; Harder et al., 2004; Bodbyl Roels and Kelly, 2011; Willmer, 2011; Liao and Harder, 2014). These traits can vary among plants and populations owing to genetic differences and plastic responses to local environmental conditions (Elle and Hare, 2002; Herlihy and Eckert, 2007; Ellis and Johnson, 2009; Morales et al., 2010; Meindl et al., 2013; Zhao and Huang, 2013; Dai et al., 2017; Christopher et al., 2021). Plasticity may underlie many of the observed phenotypic differences of *A. kusnezoffii* in contrasting habitats, as has commonly been observed for traits involved in pollination and mating (Kay and Picklum, 2013; Spigler and Kalisz, 2013; Camargo et al., 2017; Dai et al., 2017; Majetic et al., 2017; Leibman et al., 2018; Brunet and Van Etten, 2019). Two aspects of flower production are particularly suggestive of plastic responses to more favorable growth conditions in the meadows than in the forests. Most obviously, meadow genets produced 7.2× more flowers, on average, than forest genets, mostly because they grew 4.2× more ramets (Figure 1A). In addition, rather than the size‐number trade‐offs expected from resource constraint (van Noordwijk and de Jong, 1986), meadow plants produced more ramets with more and larger flowers per ramet (Figure 1A,C–E). Given these associations, we interpret the interpopulation phenotypic differences of the measured traits as primarily representing plastic responses, rather than local adaptation. A common‐garden experiment would be helpful to assess the relative extent of these influences (Turesson, 1922; Clausen et al., 1940), if it were maintained for multiple years (and generations).

All measured features of *A. kusnezoffii* phenotypes differed chiefly between the contrasting lower meadow and higher forest environments (Figure 1). Forests impose biotic and abiotic conditions on understory plants that contrast with those experienced in open environments, with diverse effects on plant phenotypes and performance (Niesenbaum, 1992; Kilkenny and Galloway, 2008; Valladares et al., 2016; Leibman et al., 2018). The small elevational differences between the meadow and forest populations (113–186 m) may also have contributed to their phenotypic differences, primarily by affecting the durations of growing and flowering seasons. *Aconitum kusnezoffii* genets in the forest populations were smaller overall (~70% fewer ramets) and invested proportionally less in reproduction than meadow plants. The latter feature is evident in forest ramets being taller, but producing smaller flowers on terminal inflorescences, and fewer lateral inflorescences with fewer flowers than meadow ramets (Figure 1B–E). Plants commonly respond to shaded conditions by reducing flower production (e.g., Niesenbaum, 1992; Kilkenny and Galloway, 2008; Cao et al., 2017; Celis et al., 2019). This effect may also have contributed to differences in flower production between populations within habitats (M1 > M2, F1 > F2: Figure 1A,C,D). Unlike population M1, population M2 was shaded for ~3 h d^–1^, and the forest occupied by population F2 had a denser canopy than that occupied by population F1. Although forest plants had fewer, smaller flowers, their flowers contained more nectar than those of meadow plants after exclusion from pollinators for 24 h (Figure 1F). This difference could reflect more soil moisture in the forests than in the meadows (see Waser and Price, 2016; Gallagher and Campbell, 2017). Overall, the observed variation illustrates differences among nearby populations in flower production, flower size, and nectar production that created contrasting foraging environments for their pollinators.

### Pollinator abundance and behavior

The differing behavior of bumble bees visiting meadow and forest genets of *A. kusnezoffii* reflects the context dependence of pollinator behavior (see Harder and Barrett, 1995; Biernaskie and Gegear, 2007) that can influence plant mating outcomes. Within plant populations, including those considered in the present study (Liao et al., 2009), bumble bees typically visit more flowers on plants with larger displays (Ohashi and Yahara, 2001). By contrast, bumble bees visited fewer ramets and fewer flowers on the larger meadow genets than on the smaller forest genets (Figure 2B,C). This contrast probably reflects differing nectar availability in the two habitats. Compared to the forest sites, meadow plants produced less nectar per flower (Figure 1F) and yet were visited by 3× more bumble bees (Figure 2A). Both features likely reduced nectar standing crop per flower of meadow plants, resulting in the briefer visits by bumble bees to their individual flowers (Figure 2D; see Hodges and Wolf, 1981; Thomson, 1986). In response to limited nectar per flower, bumble bees generally visit fewer flowers per plant (Dreisig, 2012), which suggests the proximal reason for bees visiting fewer flowers per genet in the meadow populations. According to this interpretation, contrasting conditions that determine floral‐resource productivity and consumer abundance can cause population differences in pollinator behavior that influence plant fecundity and mating (also see Brunet and Sweet, 2006; Delmas et al., 2016; Leibman et al., 2018).

### Female reproductive outcomes

Most aspects of fecundity and mating variation among the studied populations likely arose as consequences of the pollinator responses to differences in plant phenotypes. The equivalently high fruit set among meadow and forest populations (Figure 3A) is consistent with the equal probability of a flower receiving at least one visit and indicates that flowers had ample opportunity to receive some pollen, on average. By contrast, forest plants produced 12.8% more seeds per fruit than meadow plants (Figure 3B), probably owing to the longer pollinator visits to forest flowers (Figure 2D) associated with more nectar (Figure 1F; see Thomson and Plowright, 1980; Thomson, 1986).

Despite fewer seeds per fruit, meadow plants outcrossed 20.6% more than forest plants, as estimated for maternal seed families (Figure 4A, black symbols). However, this difference underestimates the incidence of cross‐ versus self‐mating, because selfed *A. kusnezoffii* zygotes experience inbreeding depression during seed development (Hao et al., 2012). Based on an estimate of pre‐dispersal inbreeding depression for population M1 (Hao et al., 2012), cross‐fertilization accounted for 27.3% more zygotes for meadow plants than for forest plants (Figure 4A, gray symbols). This difference may itself be inaccurate if the expression of inbreeding depression differed among populations owing to effects of their contrasting environmental conditions (see Armbruster and Reed, 2005; Cheptou and Donohue, 2011; Sandner et al., 2021). Nevertheless, meadow plants produced a greater fraction of outcrossed seeds than forest plants.

Because most self‐pollination in *A. kusnezoffii* involves geitonogamy (88% for population M1; Hu et al., 2015), the outcrossing difference likely primarily reflects the observed differences in the number of flowers visited per genet by individual bees, rather than within‐flower self‐pollination. In particular, greater outcrossing in meadow populations is consistent with individual bumble bees generally visiting fewer ramets and fewer flowers per genet than in forest populations (Figure 2B,C; see Harder and Barrett, 1995; Karron et al., 2004). By contrast, autogamy seems unlikely to have contributed appreciably to the habitat differences in outcrossing. Indeed, previous studies observed that male and female phases overlapped in an average of only 5% of flowers in forest population F1 during two years (Liao et al., 2009), and the outcrossing rate of intact flowers in meadow population M1 was only 1.2% lower than that of emasculated flowers (Hu et al., 2015). Regardless of the relative contributions of different modes of self‐pollination, greater pollination quality (i.e., more outcrossing) for meadow plants offset their lower per‐flower pollination quantity (i.e., fewer seeds per fruit) in relation to forest plants. Indeed, based on the product of mean seeds per fruit and mean female outcrossing rate, fruits of meadow plants produced equivalent numbers of outcrossed seeds (20.8) to those of forest plants (19.5), on average.

Despite this equivalence, forest plants mated with 14% more outcross pollen donors (Figure 4B). Because male‐mate number was assessed on the basis of seed paternity, it represents the aggregate outcomes of two sequential sets of processes. Aspects of pollination first establish maximal potential mate number, as represented by the plants that contributed cross‐pollen to a maternal plant's stigmas. Post‐pollination filtering then determines realized mate number through differential pollen‐tube germination, survival, and ovule fertilization, as well as differential zygote survival and seed development. The mixture of potential male mates in the cross‐pollen on a stigma depends on the number of contributing pollinators and the per‐pollinator and among‐pollinator diversity of their pollen loads. Given that flowers of meadow and forest plants experienced equivalent visitation probabilities, pollinator number per flower likely did not contribute to the greater male‐mate number of forest plants. In addition, per‐pollinator pollen diversity probably limited, rather than enhanced, potential male‐mate number of forest plants. Specifically, the longer flower visits and visits to more flowers per genet by individual visitors to forest plants (Figure 2C,D) should have reduced pollen carryover among genets (see Thomson and Plowright, 1980; Thomson, 1986; Hodges, 1995). Less carryover would limit donor diversity in the pollen loads carried by individual pollinators of forest plants compared to those of meadow plants (see Harder and Barrett, 1996; Mitchell et al., 2013). Instead, forest plants might have received more diverse pollen if the various pollinators that visited individual genets carried pollen from different sets of potential pollen donors (see Mitchell et al., 2005) because they followed differing paths before arriving at specific pollen‐receiving plants (e.g., Makino and Sakai, 2004). A simple model of pollen dispersal (Harder and Barrett, 1995) suggests that this mechanism could more than offset the effects of reduced pollen carryover, although this possibility needs specific examination. Compared to pollination effects, post‐pollination influences on the habitat difference in male‐mate number are less apparent, because we did not measure features of relevant processes. Nevertheless, the habitat difference reveals that mate diversity depends on both the general processes associated with outcrossing (see Pannell and Labouche, 2013) and the specific ecological characteristics that modulate mating outcomes in plant populations.

## CONCLUSIONS

Fecundity and mating systems commonly vary among angiosperm populations (Aguilar et al., 2006; Fernández et al., 2012; Whitehead et al., 2018), often in association with underlying environmental differences among sites (Barrett and Harder, 2017). Such variation is usually attributed to heterogeneous abundance of effective pollinators and its consequences for pollen limitation of fecundity and the incidence of autonomous self‐pollination (reviewed by Knight et al., 2005; Goodwillie and Weber, 2018). The results of the present study demonstrate that reproductive outcomes can also vary among populations as a result of environmental effects on plant phenotypes and their functional influences on aspects of pollinator behavior that govern pollen dispersal within and among plants. The nature of the phenotypic responses depends on the environmental features that differ among populations and their specific effects on gene expression (Sultan, 2015). The relevant pollinator responses fundamentally involve influences of plant characteristics on foraging benefits and costs, which have been demonstrated experimentally and within plant populations (Harder et al., 2001). The foraging benefits and costs also depend on environmental circumstance, including the frequency of pollinator visitation, which can differ among sites (e.g., Cuevas and Rosas‐Guerrero, 2016; Nakamura and Kudo, 2016; Parker et al., 2016; Waser and Price, 2016). The resulting context‐dependent plant‐pollinator interaction determines the local characteristics of pollinator‐mediated pollen dispersal, including overall pollen removal and receipt, the relative incidence of self‐ and cross‐pollination, and the representation of pollen from different plants on stigmas (Barrett and Harder, 2017). Thus, environmental influences on pollination and their consequences for population differences in reproductive outcomes can involve interacting effects on both plant phenotypes and pollinator behavior, in addition to variation in pollinator availability.

## AUTHOR CONTRIBUTIONS

L.D.H., D.‐Y.Z., and W.‐J.L. conceived the study. H.T., A.‐Y.W., and W.‐J.L. designed the methodology and collected the data. H.T., L.D.H., and W.‐J.L. conducted the analyses and data interpretation and wrote the manuscript. All authors gave final approval for publication.

## Data Availability

Data used in all the analyses are available through the Dryad Digital Repository: https://doi.org/10.5061/dryad.prr4xgxnm (Tian et al., 2021).
